# In Response

**DOI:** 10.4269/ajtmh.16-0246b

**Published:** 2016-07-06

**Authors:** Amanda K. Debes

**Affiliations:** ^1^ Department of International Health, Johns Hopkins Bloomberg School of Public Health, Baltimore, MD. E-mail: adebes1@jhu.edu

Dear Sir:

We thank Rebaudet and others for independently confirming the value of cholera DNA preservation on filter paper and its suitability for molecular analysis. These two studies highlight the benefits of being able to store samples at room temperature for extended periods without risk of specimen compromise, as well as the ability to transfer and ship samples without a cold-chain requirement or risk of biohazard. This reduces the need for advanced laboratory capacity in cholera-prone areas while eliminating transportation issues. Moreover, the methodology can be combined with the use of the cholera rapid dipstick test (e.g., Crystal VC, Arkray Healthcare Pvt Ltd., Gujarat, India) to provide immediate results confirmed by subsequent detailed (but delayed) molecular confirmation. We are also exploring the use of the filter paper with fecal sample directly, and agree this further simplifies the process under field conditions.

